# The surveillance of the epidemiological and serotype characteristics of hand, foot, mouth disease in Neijiang city, China, 2010-2017: A retrospective study

**DOI:** 10.1371/journal.pone.0217474

**Published:** 2019-06-06

**Authors:** Jing Li, Zeyuan Yang, Zhixuan Wang, Yong Xu, Shuibin Luo, Xuelan Yu, Juan Liu, Yan Zhou, Wenbin Tong, Peibin Zeng

**Affiliations:** 1 Neijiang Center for Disease Control and Prevention, Neijiang, Sichuan, China; 2 West China School of Public Health and West China Fourth Hospital, Sichuan University, Chengdu, Sichuan, China; 3 Sichuan Center for Disease Control and Prevention, Chengdu, Sichuan, China; Institut Pasteur, FRANCE

## Abstract

Hand, foot, and mouth disease (HFMD) is well recognized as one of the major threats to children’s health globally. The increasing complexity of the etiology of HFMD still challenges disease control in China. There is little surveillance of the molecular epidemiological characteristics of the enteroviruses (EVs) that cause HFMD in Neijiang city or the Sichuan Basin area in Southwest China. In this study, demographic and epidemiological information for 14,928 probable HFMD cases was extracted and analyzed to describe the epidemic features of HFMD in Neijiang city from Jan 2010 to Dec 2017. The swab samples of select probable HFMD cases from 2012 to 2017 were tested by reverse transcription (RT) real-time PCR to identify the serotype distribution of EVs, and 110 randomly selected RT-real-time PCR positive samples were then amplified and analyzed for the VP1 or VP4 regions of EVs to further analyze the phylogenetic characteristics of the circulating strains in this area. The eight-year average annual incidence was 49.82 per 100,000 in Neijiang. The incidence rates varied between 19.51 and 70.73 per 100,000, demonstrating peaks of incidence in even-number years (2012, 2014 and 2016). The median age of the probable cases was 27 months and the interquartile range (25^th^ to 75^th^ percentile) of ages for the probable HFMD cases was between 14 and 42 months. The male-to-female ratio of the probable HFMD cases was 1.47:1, and scattered children were the major population classification (81.7%). Two epidemic peaks were observed: one major peak between April and July and the other lesser peak between October and December. Of 6513 probable cases tested with RT-real-time PCR, 4015 (61.6%) were positive for enterovirus with the serotype distribution as follows: EV71+, 30.1% (n = 1210); CV-A16+, 28.7% (n = 1154) and a sole pan-enterovirus+, 41.1% (n = 1651). A total of 91 cases (82.7%, 91/110) were successfully amplified and underwent phylogenetic analysis: all EV71+ cases were C4a serotype (n = 23/30); all CV-A16+ cases were B2b serotype (n = 24/30); of 42 sole pan-enterovirus+ samples, 20 were CV-A6, 14 were CV-A10 and the rest within this group were CV-A4 (n = 4), CV-A8 (n = 2), CV-A9 (n = 1) and CV-B3 (n = 1). Our findings provide important evidence that aids the improvement of strategies for vaccination against HFMD and comprehensive disease control in China.

## Introduction

Hand, foot and mouth disease (HFMD) is known as an infectious disease caused by a group of enteroviruses that have a global distribution[1]. Enteroviruses are members of the genus *Enterovirus* with more than 100 serotypes and are classified into four species: EV-A, EV-B, EV-C and EV-D [2]. Enterovirus 71 (EV71) and coxsackievirus A16 (CV-A16) from EV-A are the main circulating agents that cause HFMD, and EV71 is dominant in severe cases [3]. HFMD mostly occurs in children under 5 years old, resulting in various clinical symptoms including fever, skin eruptions of hands, feet and buttocks, and mouth vesicles or ulcers [4]. In China, the incidence rate of HFMD is estimated to be 120/100,000 per year with 500–900 reported annual deaths and is frequently ranked third after tuberculosis and hepatitis on the list of notifiable infectious diseases [3, 5]. EV71 and CV-A16 are the most prevalent serotypes, accounting for more than 70% of infections reported from a Chinese national-scale investigation from 2008 to 2012, and coxsackievirus A6 (CV-A6) and coxsackievirus A10 (CV-A10) have emerged with increasing epidemics in recent years [6–8].

Neijiang city is located in the center of the Sichuan Basin in Southwest China and with a population density of 692 per square kilometers, is second only to the capital city of Sichuan Province-Chengdu, both sharing similar climate and terrain conditions [9]. Previous studies on HFMD in this region have mainly focused on the spatial-temporal factors influencing the epidemic [10–13]. There have been few reported data on the serotype distributions and profile of the molecular characteristics of enteroviruses causing HFMD. In this study, we conducted a comprehensive and updated investigation on the serotype distribution of the etiology of HFMD in Neijiang city from 2010 to 2017, which is representative of the epidemiological and molecular characteristics of HFMD in the larger area of the Sichuan Basin.

## Materials and methods

### Case definitions

According to the guidelines for HFMD diagnosis and therapy issued by the National Health Commission of the People’s Republic of China [14], HFMD cases were defined as probable cases and confirmed cases. Patients with papular or vesicular rash on hands, feet, mouth, or buttocks and with or without fever were defined as probable cases. A probable HFMD case was defined as a confirmed case if the case was positive by virus nucleic acid testing (reverse-transcription PCR or real-time PCR) or virus isolation. Confirmed HFMD cases were classified into severe cases and mild cases. Severe cases displayed severe neurological or cardiopulmonary clinical symptoms, such as acute flaccid paralysis, aseptic meningitis and pulmonary hemorrhage. Otherwise, patients were categorized into mild cases.

### Clinical and epidemiological data

From Jan 2010 to Dec 2017, a total of 14,928 probable HFMD cases from Neijiang city were reported and recorded in the China Information System for Disease Control and Prevention (http://www.chinacdc.cn). The data regarding the probable HFMD cases initially diagnosed in the seven hospitals involved in this study or confirmed in the laboratory at Neijiang CDC were extracted from the database, including demographic information, case definition (probable or confirmed), severity (mild or severe), date of onset, date of diagnosis at hospitals and virus serotype for confirmed cases.

### Sample Collection and reverse transcription (RT) real-time PCR

For the Neijiang CDC, the application of RT real-time PCR to confirm HFMD cases was enacted in Jan 2012. According to the national guidelines, throat swab samples from 6513 probable cases (available for testing out of 12751 probable cases) from Jan 2012 to Dec 2017 were collected in Neijiang city. Samples were frozen and stored at -80°C at each site before being shipped in batches to the Neijiang Center for Disease Control (CDC), Sichuan CDC and Sichuan University for further testing. Enterovirus RNA was extracted from throat swab samples using a viral RNA extraction kit (QIAamp, Qiagen, Valencia, CA) following the manufacturer’s instructions. One-step RT-real-time PCR was conducted using a commercial kit (BioPerfectus Technologies, Beijing, China; Cat No. JC20303) to detect the enterovirus RNA. The commercial assay was developed for multiple detections based on the Taqman probe method, which contains three sets of primer pairs and probes to detect EV71, CV-A16, and pan-enterovirus. The result was considered positive if amplification produced a signal at a cycle number ≤35 and otherwise was considered negative.

### Reverse transcription PCR and sequencing

Since this was a retrospective study, the samples collected before Jan 2014 were not included. From Jan 2014 to Dec 2017, randomly selected RT-real-time PCR positive samples (with amplification cycle numbers<30 during RT real-time PCR mentioned above) underwent RT-PCR and sequencing, including EV71+ (n = 30), CV-A16+ (n = 30) and a sole pan-enterovirus+ (n = 50). The viral RNAs were extracted from 140 μL throat swab samples using a viral RNA extraction kit (QIAamp, Qiagen, Valencia, CA) following the manufacturer’s instructions and eluted in 100 μL water. The 891 nucleotides of the entire VP1 region or the 420 nucleotides of the VP4 region of the virus, were amplified using the primers and procedures described previously [15, 16]. Both strands of DNA PCR products were purified and sequenced by a DNA sequencing company (BGI, Inc., Beijing, China).

### Serotyping and phylogenetic analysis

BioEdit, v7.0.4.1 (http://www.mbio.ncsu.edu/BioEdit/bioedit.html) was applied for the edit and alignment of sequences. Each complete VP1 or VP4 sequence was then compared to the sequences deposited in GenBank (https://www.ncbi.nlm.nih.gov/genbank/) using the BLAST algorithm to determine the serotype. The phylogenetic tree was built using the MEGA 6.06 tool (http://www.megasoftware.net) by the neighbor-joining method under Kimura's two parameter correction (1000 replicates). The HFMD virus reference sequences were retrieved from GenBank, including thirty-three reference sequences covering most genotypes for EV71 and CV-A16, one sequence for CV-A16 (EU812514) as an outgroup in the construction of the EV71 phylogenetic tree and one sequence for EV71 (U22521) as the outgroup in the CV-A16 phylogenetic tree.

### Statistical analysis

Statistical analysis was performed using the statistical software SAS (SAS, Windows 9.4, SAS Institute, Cary, NC; 2016). Chi-square or Fisher’s exact tests were conducted on patients’ demographic categories. P<0.05 was considered statistically significant.

### Ethics statement

The study protocol was approved by the ethics committee of West China Fourth Hospital (S1 Fig). This study was a retrospective analysis and all information were kept anonymous, so the written consents from the patients were waived.

### Gene accession numbers

The sequences of 91 HFMD virus’ VP1 or VP4 region can be retrieved using accession numbers from MK691012 to MK691102.

## Results

**Fig 1:**
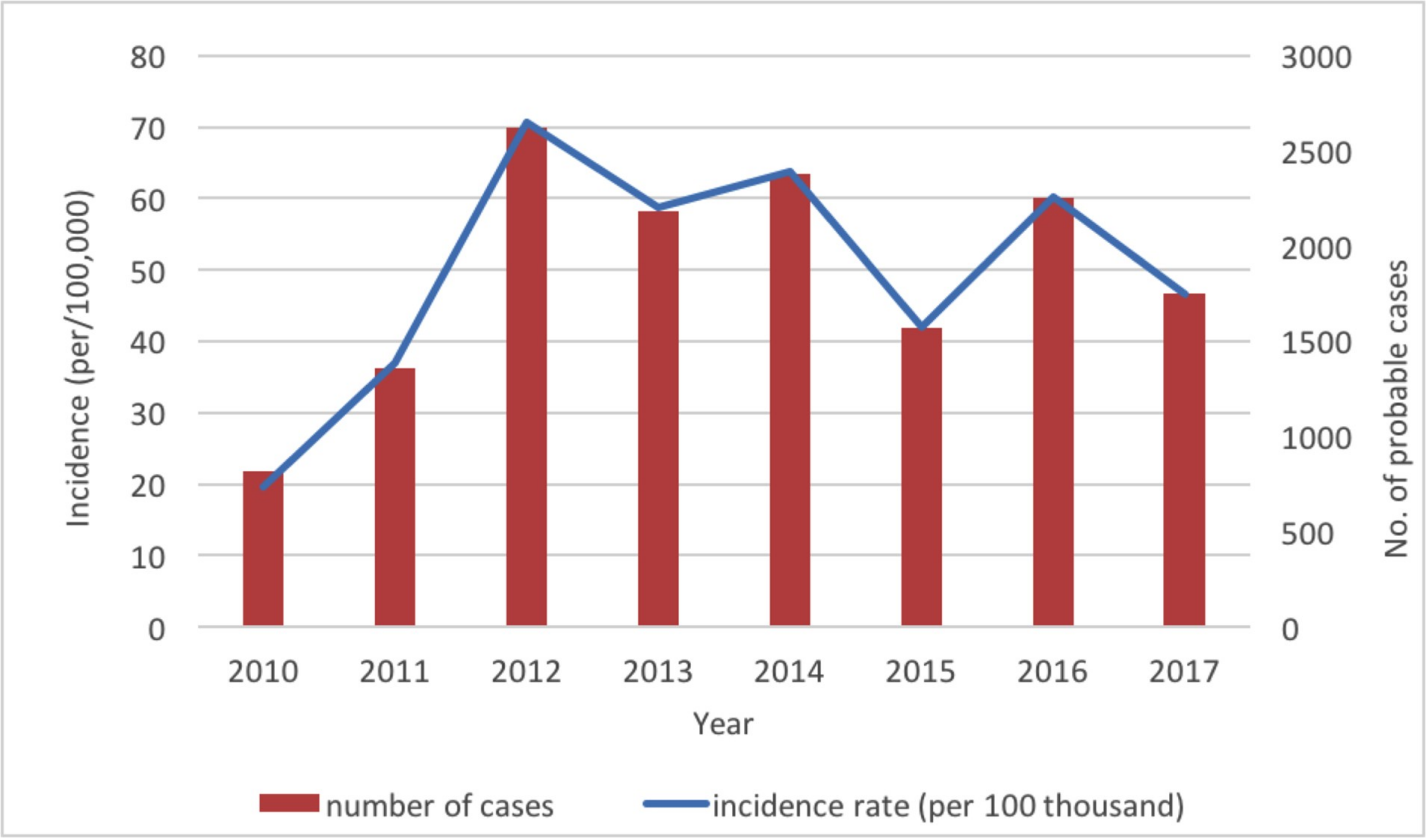
Annual incidence and number of probable cases of HFMD in Neijiang, China, 2010–2017.

**Fig 2:**
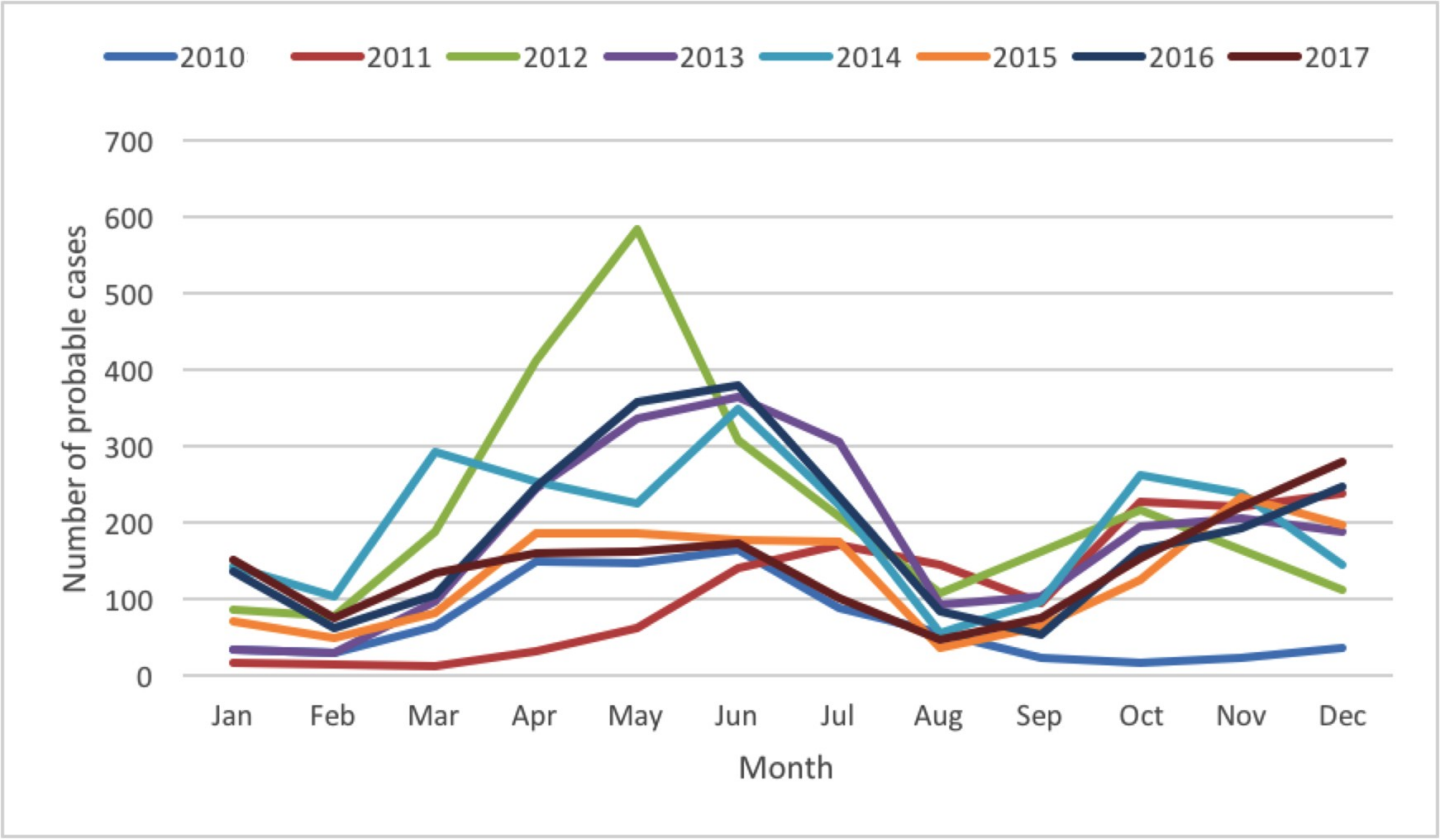
Monthly distribution of the probable HFMD cases in Neijiang, Sichuan, China, 2010–2017.

A total of 14 928 probable cases of HFMD were reported during 2010–2017 in Neijiang, China, of which 47 (0.31%) were severe cases (Table 1). The annual incidence (calculated from probable cases) increased sharply from 19.51 to 70.73 per 100,000 from 2010 to 2012 and varied between 40 and 65 per 100,000 between 2013 and 2017, with an eight-year average annual incidence of 49.82 per 100,000. Notably, the variations in the incidence rates from 2012 to 2017 demonstrated a regular pattern of sine distribution with peaks at even-number years (2012, 2014 and 2016) (Fig 1). The median age of the probable cases was 27 months (range from 1 month to 192 months). The interquartile range (25^th^ to 75^th^ percentile) of ages for the probable HFMD cases was between 14 and 42 months. There were more male than female cases (1.47:1), and most (81.7%) cases were from scattered children (children who do not attend kindergarten and are usually taken care of by their parents), followed by the children in kindergarten (16.5%). The monthly distribution of the probable HFMD cases displayed two epidemic peaks: the major peak was between April and July and the smaller peak was between October and December (Fig 2).

**Table 1:** The demographic characteristics of probable hand, foot and mouth disease cases in Neijiang, Sichuan, China between 2010 and 2017 (N, %) ^a^.

Year	2010	2011	2012	2013	2014	2015	2016	2017	In total
Incidence rate (per 100 thousand)	19.51	36.86	70.73	58.76	63.83	42	60.22	46.63	49.82
Number of probable cases	815	1362	2619	2182	2379	1572	2252	1747	14928
Number of severe cases ^b^	2	14	0	12	6	3	7	3	47
Age (months)									
Median	28	23	26	32	30	24	34.5	27	27
IQR ^c^ (25^th^ to 75^th^ percentile)	12–38.5	15.5–36	13–41.5	18–46	17–42	15–46	19–48.5	14–42	16–39
Gender									N = 8765^d^
Male	538(66.0%)	N/A^d^	N/A	N/A	1400 (58.8%)	945 (60.1%)	1339 (59.5%)	996 (57.0%)	5218 (59.5%)
Female	277(34.0%)	N/A	N/A	N/A	979 (41.2%)	627 (39.9%)	913 (40.5%)	751 (43.0%)	3547 (40.5%)
Population classification									
Scattered children ^e^	511 (62.7%)	1051 (77.2%)	2072 (79.1%)	1866 (85.5%)	2111 (88.7%)	1359 (86.5%)	1842 (81.8%)	1380 (79.0%)	12192 (81.7%)
In kindergarten	276 (33.9%)	269 (19.8%)	459 (17.5%)	294 (13.5%)	238 (10.0%)	199 (12.7%)	381 (16.9%)	342 (19.6%)	2458 (16.5%)
In primary school	24(2.9%)	27(2.0%)	51(1.9%)	19(0.9%)	26(1.1%)	10(0.6%)	23(1.0%)	21(1.2%)	201(1.3%)
others	4(0.5%)	15(1.1%)	37(1.4%)	3(0.1%)	4(0.2%)	4(0.3%)	6(0.3%)	4(0.2%)	77(0.5%)

^a^ Data are reported as the number and percent of total in each year and in total.

^b^ No fatal case was reported within severe cases.

^c^ IQR: interquartile range.

^d^ The gender data between 2011 and 2013 were not available; N/A, not available.

^e^ Scattered children are defined as children who were taken care of by their family members

**Fig 3:**
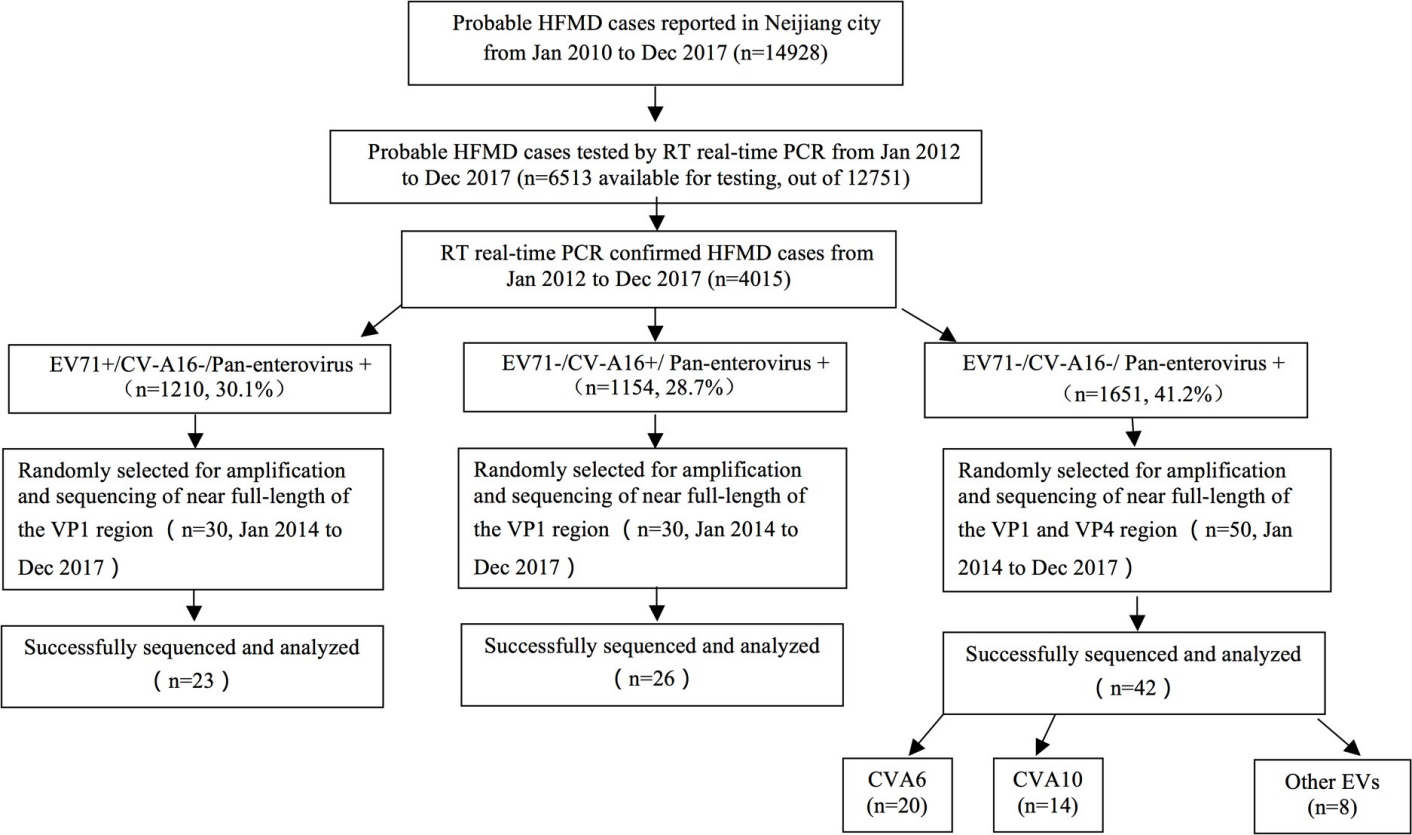
Flow diagram of the epidemiology and genotype investigation of HFMD patients. EV-A71 = enterovirus A71. CV-A16 = coxsackievirus A16. *Classification according to the criteria issued by National Health and Family Planning Commission of the People’s Republic of China.

**Fig 4:**
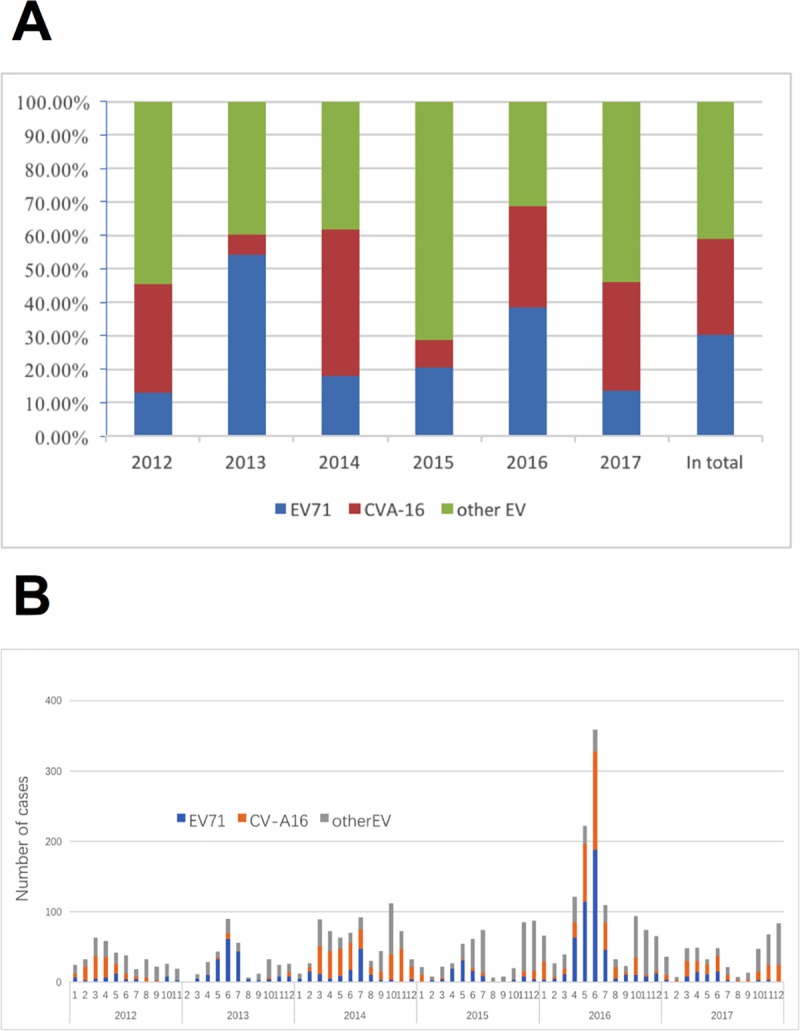
The yearly and monthly distribution and constituent ratio of the enterovirus serotype associated with confirmed HFMD cases in Neijiang, Sichuan, China, 2012–2017. A) Yearly distribution; B) Monthly distribution.

Of the 4015 probable cases confirmed by RT real-time PCR and identified for virus serotype, the distribution of EV71, CV-A16 and other EVs were 30.1% (n = 1210), 28.7% (n = 1154) and 41.1% (n = 1651), respectively (Fig 3). From 2012 to 2017, the serotype distributions changed frequently, and the proportion of EV71 varied between 13.1% (2012) and 55.5% (2011), and CV-A16 varied between 6.1% (2013) and 43.7% (2014), while other EVs varied between 12.9% (2011) and 71.3% (2015) (Fig 4A). The monthly distribution and constituent ratio of enterovirus serotype associated with HFMD confirmed cases was displayed in Fig 4B.

**Fig 5:**
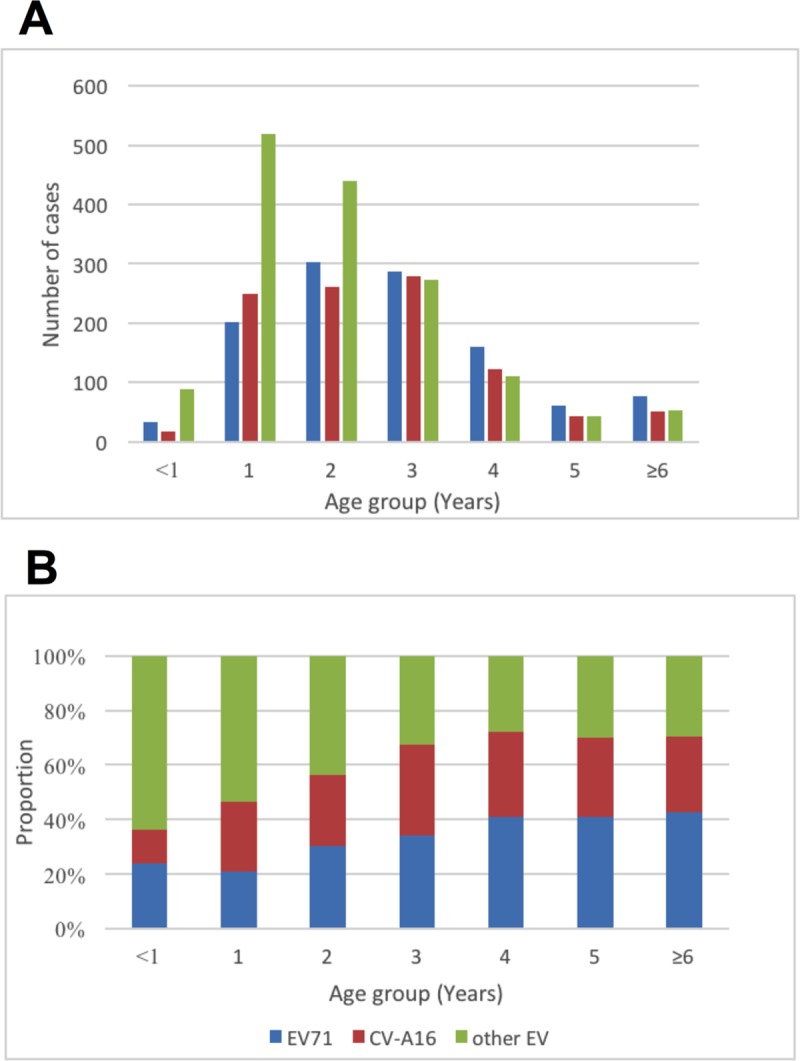
Enterovirus serotype distribution by age group in confirmed HFMD cases in Neijiang, Sichuan, China, 2012–2017.

The serotypes of “other EVs” were more frequently observed in children under 2 years of age, and the proportion of “other EVs” decreased significantly with age from 64.1% to 29.4%. Conversely, EV71 and CV-A16 increased with age: EV71 increased from 23.7% to 42.8% and CV-A16 increased from 12.2% to 27.8%. The distribution of the three groups of serotypes (EV71, CV-A16 and “other EVs”) became stable in children aged 3 years and older (Fig 5).

**Fig 6:**
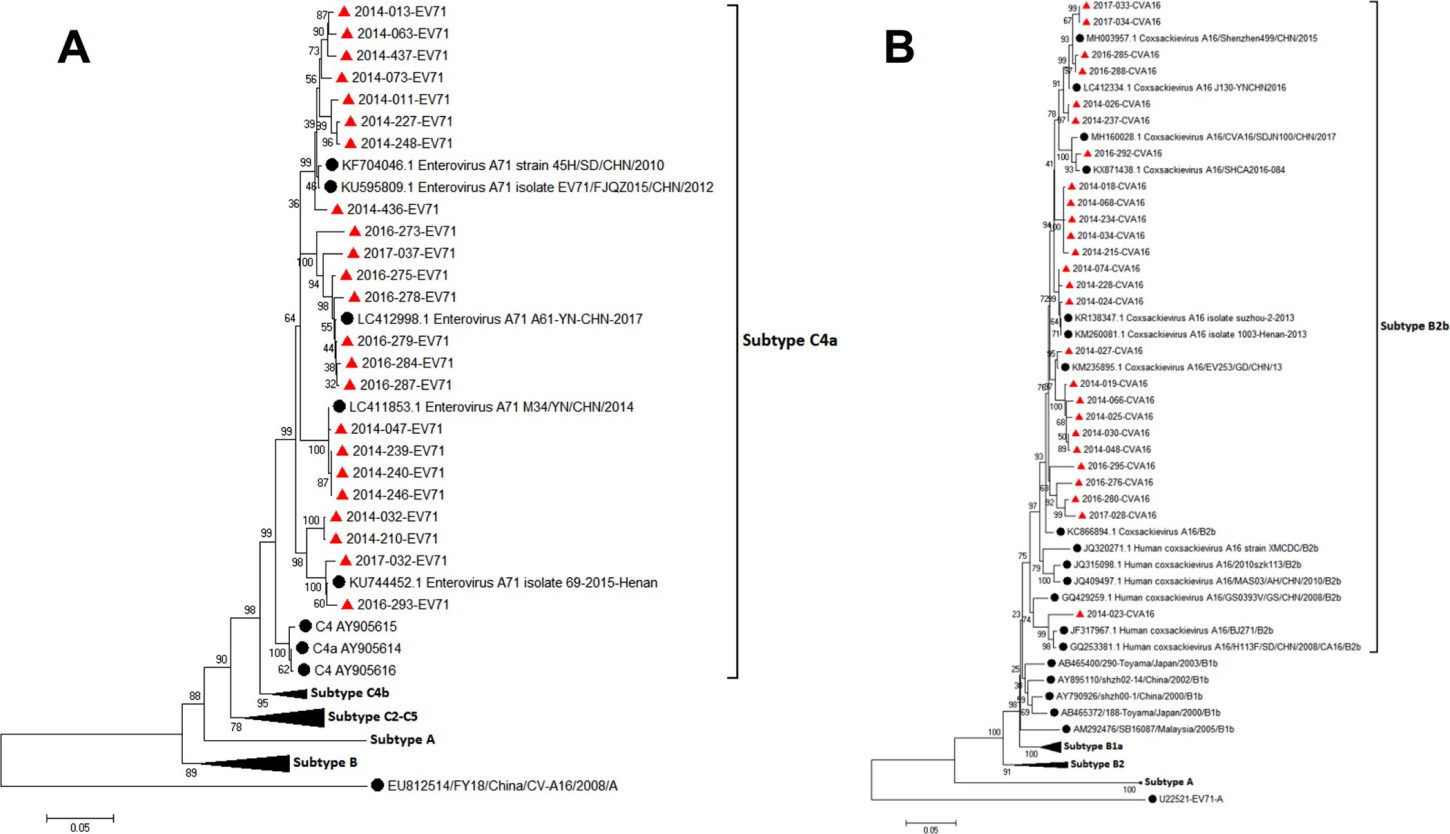
Phylogenic tree of EV71 (n = 23) and CV-A16 (n = 24) from Neijiang city. A) EV71; B) CV-A16; Red triangle, sequences from Neijiang city; Black sphere, reference sequences.

Of 110 randomly selected RT-real-time PCR positive samples, RNA from 91 cases was successfully amplified and analyzed for the VP1 or VP4 regions: EV71+ (n = 23/30), CV-A16+ (n = 26/30) and a sole pan-enterovirus+ (n = 42/50) (S1 Text). For the entire group, all EV71+ cases were C4a serotype (by VP1 genotyping) (Fig 6), while all CV-A16+ cases were B2b serotype (by VP1 genotyping). Of the 42 sole pan-enterovirus+ samples, 20 were CV-A6 (by VP4 genotyping), 14 were CV-A10 (by VP1 genotyping) and the other EVs identified by VP4 genotyping (n = 8) within this group were CV-A4 (n = 4), CV-A8 (n = 2), CV-A9 (n = 1) and CV-B3 (n = 1).

## Discussion

Based on the data of an eight-year (2010–2017) surveillance of HFMD cases in Neijiang city, our study provided a comprehensive review of the epidemiology and distribution of HFMD serotypes in this area. The estimated incidence of HFMD was 49.82 per 100,000, which was lower than the national average of 120 per 100,000 (2008 to 2012) [3] and the adjacent Chongqing city (also located on inner land in Southwest China with an incidence rate of 114.8 per 100,000 in 2009–2016) [17]. The incidence rate was similar to that from the Sichuan provincial investigation from 2008 to 2013 (43.65 per 100,000)[12]. The incidence rates between 2012 and 2017 represented a temporally changing epidemic in Neijiang, similar to the annual epidemic in Japan, but different from other provinces in China[17, 18], Taiwan[19], Singapore[20], and Malaysia[21], where a recurring epidemic was observed every 3 to 4 years. The seasonal pattern of the HFMD epidemic in Neijiang during the study period showed two main epidemic peaks: one in late spring and early summer and the other in late autumn and winter, which was close to that in the Chongqing region [17]. This seasonal pattern is consistent with the large-scale national analysis from 2008 to 2012[3]. Factors driving the epidemic and seasonal pattern may be related to geographic locations, population density, environmental conditions and even the carriers of the virus, which need to be further evaluated.

The age profile suggests that most infected cases were between 14 and 42 months, similar to the reports from other Chinese regions and other countries [3, 6, 17, 19, 22–24]. Several studies have described that more infections were observed in males than in females, which was also found in our study. One possible explanation was that male children may have a higher risk of exposure to enteroviruses due to behavior patterns, such as more frequently touching unclean toys and facilities. Additionally, the different susceptibilities at the level of host immune status between males and females may contribute to this sex ratio discordance [3]; however, this presumption requires more experimental evidence.

A high proportion of scattered children among the probable HFMD cases was found in Neijiang during the study period. This is probably because most children in this study were under 3 years of age (median age: 27 months with IQR between 16 and 39 months), and there is a mandatory requirement that the children entering public kindergartens are older than 3 years of age in China. In addition, although private kindergartens do not have the age limitation for entrance, very few children under 3 years of age go to kindergarten for possible reasons such as a higher expense for private than public kindergartens, a lack of trust from parents for private kindergartens, and less availability and accessibility of private kindergartens. Even in the developed regions of China, such as Shanghai, the estimated number of annual newborn babies in this area is approximately 250,000, but only 6,000 children under 3 years old go to private kindergartens [25].

In our study, 4015 HFMD cases analyzed by RT real-time PCR demonstrated that the serotype distribution of the three classifications was EV71 (30.1%), CV-A16 (28.7%) and other EVs (41.2%). The profile of the predominant serotype with other EVs was similar to that from Chongqing city, where the rate of other EVs was as high as 63.9% [2], but different from other regions in China where EV71 was the leading serotype [3, 6, 26]. Variations in the serotype/genotype distribution in different Chinese geographic locations can be commonly found with other viruses, such as HIV [27], HBV [28] and HCV [29]. This is probably because of the diversity of transmission routes and different levels of susceptibility to viruses within the population. It can be inferred that a stable population of HFMD virus has not been formed in the Neijiang region due to the annual fluctuation of the serotype proportions during the study period. In addition, the VP1 sequences of EV71 and CV-A16 from Neijiang were merged in subtypes C4a (EV71) and B2b (CV-A16), which are commonly found in China and have a close phylogenetic relationship with those from other Chinese regions, and no distinct regional cluster was observed. The transmission routes and circulating pattern of HFMD virus in this area should be further explored.

Vaccination against EV71 was initiated in 2015 in China [30], which might be expected to reduce the fatality rate of HFMD because over 80% of severe cases and 90% of deaths of HFMD were from EV71 infections[3]. However, EV71 only accounted for 30.1% in our study, similar to neighboring Chongqing with 25.1% [2]. Since the inactivated monovalent EV71 vaccines offer no protection against HFMD caused by CV-A16 and CV-A6 and CV-A10 are emerging as prominent new serotypes among the classification of other EVs [7, 8, 26, 31](20/42, CV-A6; and 14/42 CV-A10 by subtyping from Neijiang city), the polyvalent vaccine against the HFMD virus would be urgently needed to enlarge the coverage range protected by vaccinations.

The limitations of this study were that only 51.1% (6513/12751) of probable HFMD cases were included in the analysis of the serotype distributions of the three main classifications, and only 110 cases selected from 2014 to 2017 were subtyped to identify the molecular characteristics of the HFMD virus. Further studies should focus on including more probable HFMD cases for RT real-time PCR and sequencing to obtain a comprehensive etiological distribution in this area. However, we can preliminarily obtain the sketch of the serotype composition and a glimpse of the phylogenetic relationships of the EV71 and CV-A16 strains in Neijiang associated with those from other regions in China. Additionally, the impacts of meteorological factors and variations in districts should be included in further analysis to better understand the epidemic patterns of HFMD in this area.

## Conclusions

In conclusion, our findings depicted the HFMD epidemic status and serotype distributions in the Neijiang region and contributed to the databank of comprehensive HFMD control and prevention in China.

## Supporting information

S1 Fig The ethnics statements approved by the ethics committee of West China Fourth Hospital. (TIFF)

S1 Text The 91 enterovirus sequences from Neijiang city. (TXT)

S1 Data (XLSX)
