# Correction: The surveillance of the epidemiological and serotype characteristics of hand, foot, mouth disease in Neijiang city, China, 2010-2017: A retrospective study

**DOI:** 10.1371/journal.pone.0219726

**Published:** 2019-07-10

**Authors:** The PLOS ONE Staff

The Data Availability statement for this paper is incorrect. The correct statement is: A de-identified dataset is provided in the Supporting Information files. Please contact the corresponding author Peibin Zeng (zengpeibin@live.cn).

The Funding statement for this paper is incorrect. The correct statement is: The study was supported by the Key Research and Development Project, Department of Science and Technology, Sichuan Province (Grant No. 2018SZ0212), the Fundamental Research Funds for the Central Universities. The funders had no role in the study design, data collection and analysis, decision to publish, or preparation of the manuscript. The publisher apologizes for the errors.
