# Supplementary figures and images for: The surveillance of the epidemiological and serotype characteristics of hand, foot, mouth disease in Neijiang city, China, 2010-2017: A retrospective study
Source: PLoS One. 2019 Jun 6;14(6):e0217474. doi: 10.1371/journal.pone.0217474 (PMC6553746; doi:10.1371/journal.pone.0217474)

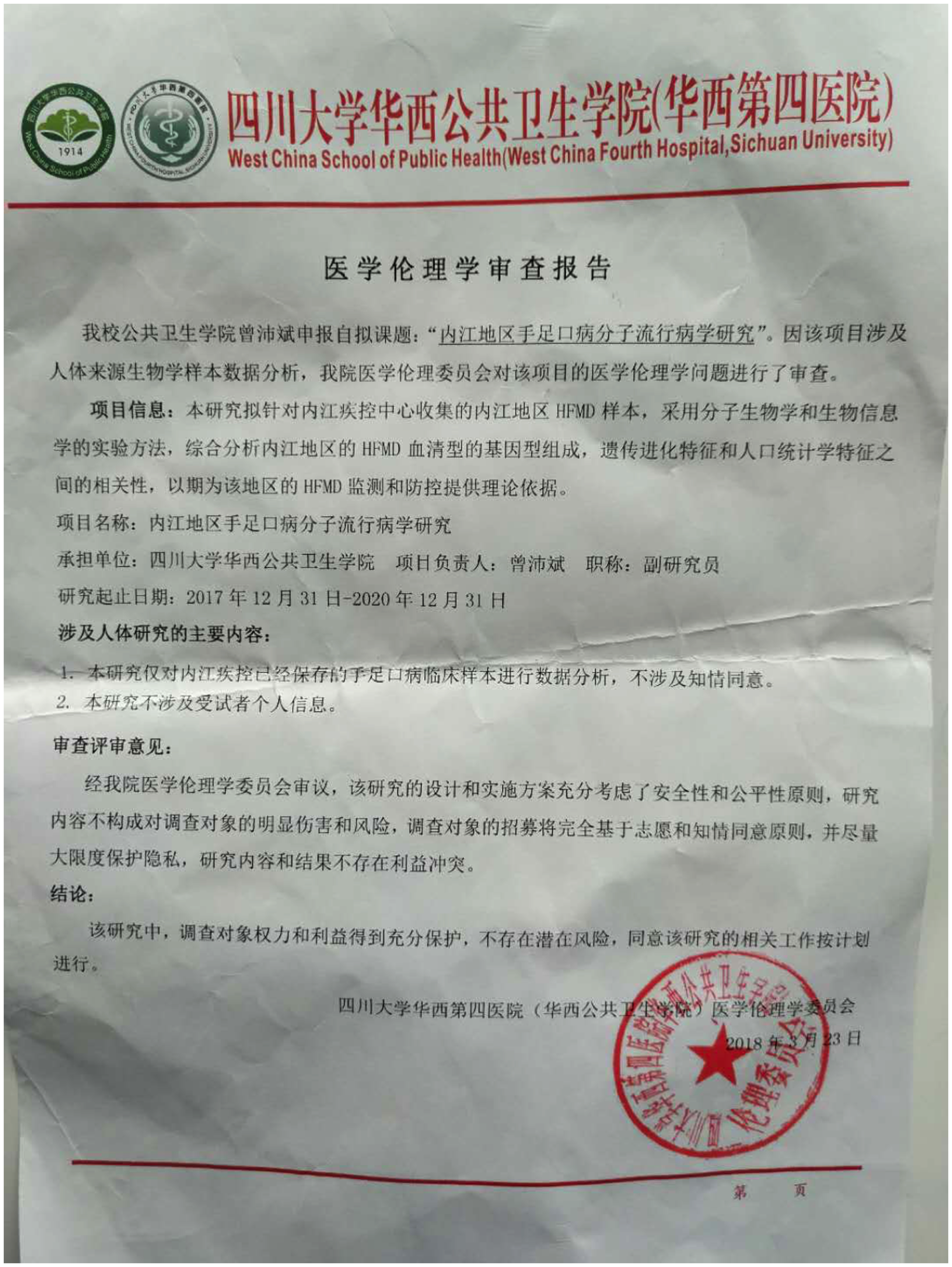

Supplement: S1 Fig — (TIFF) [file pone.0217474.s001.tiff]
